# Bridge Damage Identification Using Deep Neural Networks on Time–Frequency Signals Representation

**DOI:** 10.3390/s23136152

**Published:** 2023-07-04

**Authors:** Pasquale Santaniello, Paolo Russo

**Affiliations:** DIAG Department, Sapienza University of Rome, Piazzale Aldo Moro 5, 00185 Rome, Italy; santaniello.1888996@studenti.uniroma1.it

**Keywords:** structural health monitoring, deep learning, vibrational damage detection, synchrosqueezing transformation, feature extraction

## Abstract

For the purpose of maintaining and prolonging the service life of civil constructions, structural damage must be closely monitored. Monitoring the incidence, formation, and spread of damage is crucial to ensure a structure’s ongoing performance. This research proposes a unique approach for multiclass damage detection using acceleration responses based on synchrosqueezing transform (SST) together with deep learning algorithms. In particular, our pipeline is able to classify correctly the time series representing the responses of accelerometers placed on a bridge, which are classified with respect to different types of damage scenarios applied to the bridge. Using benchmark data from the Z24 bridge for multiclass classification for different damage situations, the suggested method is validated. This dataset includes labeled accelerometer measurements from a real-world bridge that has been gradually damaged by various conditions. The findings demonstrate that the suggested approach is successful in exploiting pre-trained 2D convolutional neural networks, obtaining a high classification accuracy that can be further boosted by the application of simple voting methods.

## 1. Introduction

Large-scale infrastructures have seen accelerated aging and deterioration of functionality in the current era of urbanization and climate change. The structures’ operational states have frequently been disrupted as a result of rising population and traffic, unanticipated natural disasters, and human-caused damage, in addition to countless instances of catastrophic breakdowns. In the context of civil engineering, damage can be defined as a change in a system’s geometric or material properties that has a negative impact on its performance, safety, dependability, and operational life [1]. This definition states that damage does not always translates into a system total failure, but rather a relative decline in system functionality leading to subpar performance [2]. Furthermore, the damage may build up until it reaches the failure state if no corrective action is done. Depending on the type of damage, systems may fail suddenly or gradually [3].

Different system damage detection (SDD) techniques are available depending on their data analysis approach. Visual inspections are the foundation of traditional methods for diagnosing damage to civil constructions. However, a number of issues prevent the practical adoption of these approaches [4]. The size of civil structures is generally quite vast, making routine inspection tedious, time-consuming, and costly. Moreover, because traditional methods rely on human judgment, experienced and highly trained labor is unquestionably needed. Finally, some structural defects are difficult to detect, even for human experts, resulting in unnoticed damages until the structure becomes impaired. In order to overcome these issues, a promising approach is to rely on vibration-based SDDs [5]. These systems ultimately aim to overcome the issues associated with the conventional SDD approaches by providing a methodical, practical, and consistent way of identifying the presence, as well as locating and quantifying the severity, of the structural damage based on the vibration response of the monitored structure. Moreover, vibration-based SDDs can be divided into *time domain* and *frequency domain* methods. The former relies only on a system’s temporal properties, usually by taking as input the raw or filtered data coming from physical sensors distributed on the structure. These approaches are conceptually simpler to use, but, because they are more susceptible to noise contamination and environmental influences, they show significant performance issues for large buildings [6]. In fact, phase shifts between the vibration responses of the undamaged and damaged structures occur in the time domain as a result of changes in natural frequencies brought on by damage. In order to obtain this information, response functions of both damaged and undamaged structures are subtracted from one another, which results in a beating phenomenon in the presence of a frequency mismatch. Even so, meaningful findings can only be obtained if the experimental hardware is kept constant during the whole study. This recommends the usage of smart structures that can both actuate and monitor the vibrations of the host structure using piezoceramic patches that are surface mounted on, or implanted in, the structure [7]. On the other hand, frequency domain techniques exploit a system’s frequency properties. Although discrete variations in the natural oscillation frequency might not be enough to uniquely identify the damage location, changes in structural frequencies may be a reliable indicator of the existence of damage [8]. In order to correlate analytical data with experimental data, frequency-domain-related damage detection algorithms often retrieve damage characteristics by modifying an analytical reference model. Unfortunately, computational inaccuracies are often introduced because the analytical model can only approximate the behavior of a real mechanical structure [9]. Some of the existing research, such as [10,11], shows that, in the field of frequency-based damage identification, either a small subset of the first few model frequencies or all frequencies recorded during measurement are used for damage detection. Nevertheless, the field is plagued by a lack of systematic investigations for choosing modal frequencies, which could be a better tool for identifying damage in practical applications, where errors or mismatches between experimental and numerical analyses cannot be disregarded since they are sensibly affected by data noise. Finally, time–frequency (TF) algorithms [12] are a promising way to have the best of both worlds because, in contrast to pure time or frequency domain approaches, they can recognize both the frequency components of the signal and their time-variant features, resulting in efficient and valuable tools to extract structural health information. Short-time Fourier transform (STFT) [13] and Wigner–Ville distribution [14] are two common TF techniques. The wavelet transform (WT), which divides a signal into a number of local basis functions, is the most popular TF technique [15].

As a way to enhance the identification of time-varying parameters of structures, synchrosqueezing transform (SST), a promising and flexible WT, is investigated in this article. SST, as opposed to standard TF techniques such as STFT or continuous wavelet transform (CWT) that do not take advantage of the signal sparsity, adds a nonlinear post-processing mapping to a conventional STFT or CWT representation. The mapping results in a condensed and sparse TF representation of the signal, pushing the energy content into the STFT’s most noticeable frequencies [16]. Any structure’s sensitivity to change with any modification in the structural attributes determines its damage characteristics in relation to its modal frequency. In this context, machine learning (ML) approaches can be successfully used to discriminate between damaged and undamaged structures, thanks to recent advances in both the developed algorithms and computational power. These techniques include, but are not limited to, convolutional neural networks (CNNs), support vector machines, and self-organizing maps, among others. In general, those methods can be applied with success to both time domain and frequency domain data. However, these approaches rely on the availability of structured and consistent data, often performing poorly if the input data are noisy or redundant, or if the training set is not big enough to generalize. In this paper, an improved SST paired with a deep learning approach is suggested in order to perform damage detection on bridge structures. In particular, a pipeline with different splitting methods is provided, in order to boost the classification power of a neural network fed with RGB images obtained by applying a colormap on SCWT data. Our intent is to give a general method for dealing with vibration damage detection tasks, starting with the sensor’s acquirements, which could also take advantage of pre-trained deep models without the need to train them from scratch. In fact, in this study, we demonstrate that pre-trained deep CNNs, fine-tuned on images representing the time-frequency content of the signal data, are actually able to extract important features useful for damage classification. The rest of the paper is organized as follows. Section 2 contains a description of the most successful methods for vibration damage detection, in order to give important insights into the work carried out so far in this field. In Section 3, we show the proposed method, analyzing its pipeline and main characteristics. In Section 4, we report on the hyperparameters and general settings of the conducted experiments, as well as report on the considered benchmark dataset. In Section 5, we show the results obtained by our method while performing a comparison with other available techniques, while in Section 6 we report the final considerations on our method and possible future works.

## 2. Related Works

The traditional method of structural monitoring (visual inspection) entails hiring a qualified structural inspector to examine the building, spot problems, and put proper maintenance plans in place. Although arduous, subjective, and prone to error, this kind of manual structural inspection uses up a significant portion of the annualized maintenance budget. In order to overcome the issues related to manual visual inspection, structural health monitoring (SHM) offers a sensor-driven real-time inspection method [17]. Based on the collected data, vibration-based SHM approaches provide realistic solutions for tracking the time-varying behaviors of aging buildings. Instrumentation and data collection, condition evaluation, damage detection, and damage prognosis are the four main components of vibration-based SHM. The structures are initially equipped with a variety of sensors to gather useful measurements (such as acceleration and displacement). Using various system identification and damage detection techniques, the gathered data are then evaluated to determine the structure’s state and spot any changes. Then, SHM exploits a variety of prognosis approaches, maintenance, and retrofitting solutions to determine the remaining structure’s usable life and the actions required to enhance its structural condition. Most vibration data are typically gathered via a dense network of wired sensors placed throughout the structure. However, because installing cables requires much effort, they are not a practical or affordable solution for towering buildings or long-span bridges. For this reason, smart wireless sensors have been proposed as a solution to the drawbacks of wired sensors [18]. Another possible solution is to rely on test vehicles equipped with sensors to ease the process of data acquisition, with promising results [19].

Recently, SHM has been accomplished by the analysis of images and videos taken by modern sensors such as cameras, robotic sensors, telephones, and drones [20]. Regardless of the type of sensor, the accuracy of the existing damage diagnosis and localization algorithms heavily depends on the accessibility of several high-quality sensors and datasets. This is a significant barrier that often prevents the application of SHM on big structures such as buildings and bridges. In order to identify changes in the vibrational qualities of modal parameters (such as frequency, damping, and mode shapes) or physical parameters (such as stiffness, damping, or mass), the essential idea underlying vibration-based damage detection (VDD) systems is to use pattern recognition algorithms [21]. Any modification of these characteristics through time can be potentially correlated to structural damages. These techniques are simpler to use, but they pose problems for large construction projects because of noise pollution and environmental issues [22]. Moreover, although discrete variations in natural frequencies can in principle be used to pinpoint the precise position of structural damage, in practice this may not always be the case because a crack in two separate places could have the same frequency variation regardless of where it is. Time–frequency methods [23] can recognize the signal frequency components and comprehend their time-variant properties, in contrast to time-only and frequency-only domain techniques. The hidden information in the data that is missed by stand-alone temporal or frequency domain approaches can be found and tracked using TF methods applied on VDD [24]. Short-time Fourier transform is a popular version of Fourier transform that enables the investigation of nonstationary signals in the TF domain (STFT) [25]. The Fourier transform of a fixed windowed signal serves as the basis for STFT. Only a small portion of the signal is examined by this windowing technique at each time step t, after which a 2D signal depending on time and frequency components is obtained. Moreover, the time–frequency resolution of the STFT technique is inversely correlated with window length. While lengthening the window improves frequency resolution, it also hinders the representation’s ability to monitor frequencies. Due to the fact that the chosen window size is identical for all frequencies, one of the greatest disadvantages of STFT is that a high resolution in both time and frequency cannot be simultaneously achieved [26]. By generalizing the connection between a nonstationary, time-variant process power spectrum and autocorrelation function, the Wigner–Ville distribution can be obtained [27], as a signal can be represented in a high-resolution TF space using the Wigner–Ville distribution.

Another way to frame VDD is as a pattern recognition task. In fact, pattern recognition-based VDD algorithms’ main goal is to identify patterns in the features of damaged and undamaged structures under the same operational and ambient conditions. Different time-series modeling and machine learning methods have been applied to pinpoint the crucial VDD properties [28]. Time-series modeling is one of the most commonly used techniques for identifying structural degradation. The fundamental steps of this approach are the creation of a time-based model, the assessment of model coefficients, and the computation of residual errors; any divergence in the coefficients or residual errors might be interpreted as a structural damage. Another popular VDD technique is based on auto-regressive (AR) models. The AR models make the assumption that the observations contain noise and thus sample their modeling error from a Gaussian distribution. Several variations of the AR models, such as the auto-regressive 15 moving-average (ARMA) [29], the AR-integrated moving-average (ARIMA) [30], and the AR model with exogenous input (ARX) [31] are widely employed in SHM as well as for damage detection. Moreover, the Mahalanobis distance, when applied to time-series pattern recognition, has demonstrated encouraging results, for instance, when [32] tested several distance metrics for damage identification. Finally, out of the several available ML methods, other popular approaches such as artificial neural networks (ANN) [33], support vector machines [34], random forests [35], and clustering techniques [36] have been extensively employed in the VDD field, with reviews that deepen their usage for civil structure health monitoring [37].

Traditional VDD approaches are based on the stationarity assumption of the vibration response and selection of modal orders. However, these approaches have trouble identifying the nonstationary component of vibration response that comes from natural hazards. A structure’s intrinsic flaws also contribute to the frequency-dependent nonstationarity of the response, along with excitation-induced nonstationarity. In fact, when there is an amplitude- and frequency-dependent nonstationary response, it becomes much more difficult to identify the damage [38]. This problem can be handled by employing sophisticated TF techniques. WT is an enhancement over-fixed window-based STFT and offers the fundamentals of the conventional Fourier transform with flexible window placement and size [39]. It can be divided into discrete and continuous wavelet transform (CWT) [40], and provides the flexibility of combining high time and frequency resolutions with a suitable basis function. Many VDD applications, including signal noise filtering, data compression, and pattern recognition, use the CWT signal processing technique. To identify sudden changes in a time-variant system, a wavelet-based frequency response function [41] has been adopted. A suitable approach is to first process the signal with a CWT, and then employ the generalized discrete Teager–Kaiser energy operator to locate and magnify the modes of a damaged structure [42]. More in detail, in the previous work the operational deflection forms of the structure were also obtained using a state-of-the-art method that applied joint approximation diagonalization of the power spectral density matrices. However, CWT and CCWT require a significant increase in frequency resolution to detect minute damage because of sensitivity issues when dealing with minute frequency changes in the structures.

Another possible way to perform damage detection is to rely on deep learning algorithms trained in a supervised setting. In fact, for the establishment of a statistical model during the training phase, supervised learning methods need labeled data for both undamaged and damaged categories. The identification, classification, and quantification of the damage are usually carried out using DL techniques such as CNNs, fully convolutional networks (FCNs), or recurrent neural networks (RNNs) in VDD-based literature. For effective bridge SHM, a sparse coding-based CNN method with wireless sensors was investigated [43]. In order to extract high-level features from acceleration data, sparse coding was applied as an unsupervised layer for unlabeled data. For a three-span bridge that was instrumented using wireless sensors, several levels of damage situations were taken into account. The proposed method outperformed previous techniques such as logistic regression and decision trees, with a final accuracy equal to 98%. Another possibility is to identify structural degradation directly using CNNs [44]. To find the best CNN settings, 50 network topologies with different hyper-parameters were examined. Recently, the use of autoencoder data compression and a one-dimensional (1D) CNN for anomaly detection in a lengthy suspension bridge has been proposed [45]. Moreover, a CNN-based VDD technique for damage identification in compressed data has also been proposed [46]. In a following study [47], a 1D CNN technique was employed on three structural assemblages: an iron girder, a short steel beam bridge, and a long steel viaduct bridge to detect changes in material properties. Finally, an alternative approach [48] has been developed, in which the time history of a vibration signal can be given as input directly into CNNs, requiring only basic array operations and a shallow architecture with fewer hidden layers. All these methods are characterized by the use of a 1D CNN trained from scratch. In order to exploit the expressive power of pre-trained CNNs, the vibration data need to be transformed into images by using time–frequency transformations; the generated colormap can be then used as input for a pre-trained 2D CNN [49]. In order to provide the necessary labeled data, a possible solution is to simulate a scaled-down bridge model [50], while exploiting a 2D-CNN architecture that reaches up to 97% accuracy while being able to distinguish damage from structurally symmetrical locations. A recent work [51] successfully applied a TF transformation on signals coming from a real railway bridge that underwent a retrofitting procedure to perform an anomaly detection task. Other approaches with their pros and cons are reviewed in a survey paper [52].

## 3. Proposed Methods

**Figure 1:**
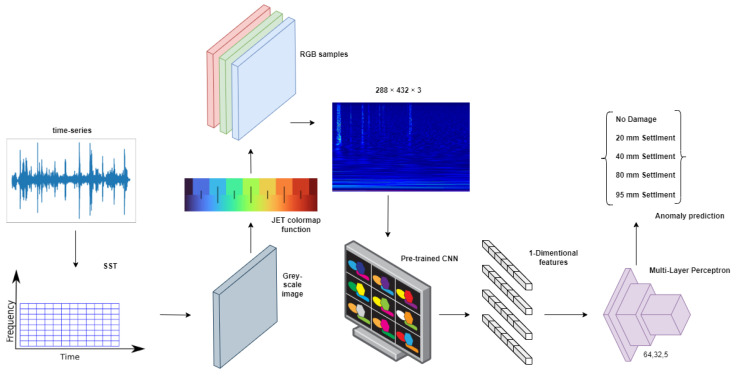
Pipeline of the baseline proposed method. The input signal is transformed by the SCWT function to a 2D gray-scale image and then expanded into an RGB format through the use of the JET color mapping. The resulting image is fed to the backbone network (pre-trained CNNs), which produces the visual features exploited by the fully connected layer for the classification.

In this work, we propose a pipeline working on hybrid time–frequency-transformed data in order to solve a multiclass classification task in which different categories correspond to different damage scenarios applied to a bridge. The baseline architecture of our method is represented in Figure 1. The raw data are composed of a time series representing accelerometer measurements in the time domain. In order to maximize the classification accuracy on the test set, we provide different methods based on visual features extraction of RGB images calculated on the provided data. Such images are obtained using the synchrosqueezing continuous wavelet transform function [53] (SCWT), which produces a 2D time–frequency signal representation that can be plotted as a gray-scale image. In order to apply the most modern and competitive CNN techniques, we transform the image from gray-scale to the RGB domain by using a JET colormap function, and use such obtained images to extract visual features from the last convolutional layer of a CNN. In order to enhance the performance of the feature extraction step further, we propose two feature-enhancing techniques. The first proposed method, based on image-splitting, focuses on cropping the input image to obtain more features for classification. The second method, based on an signal-splitting mechanism, focuses on exploiting with greater detail the spatial relationship in the time domain by splitting the signal into several pieces and calculating the SCWT for each of them. Both techniques proved to be successful in increasing the base pipeline accuracy, as demonstrated in Section 5.

### 3.1. Data Pre-Processing

**Figure 2:**
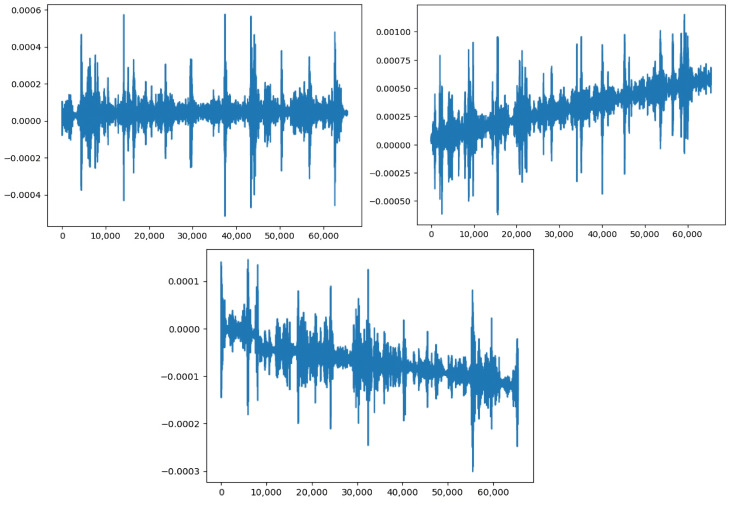
Time domain representation of 1D input signals taken from the considered dataset.

As we transform time-series measurement signals, represented in Figure 2, into images that will serve as input for our pipeline, a critical step is the choice of the function responsible for the continuous wavelet transform, as it must ensure the generation of expressive time–frequency images.

**Figure 3:**
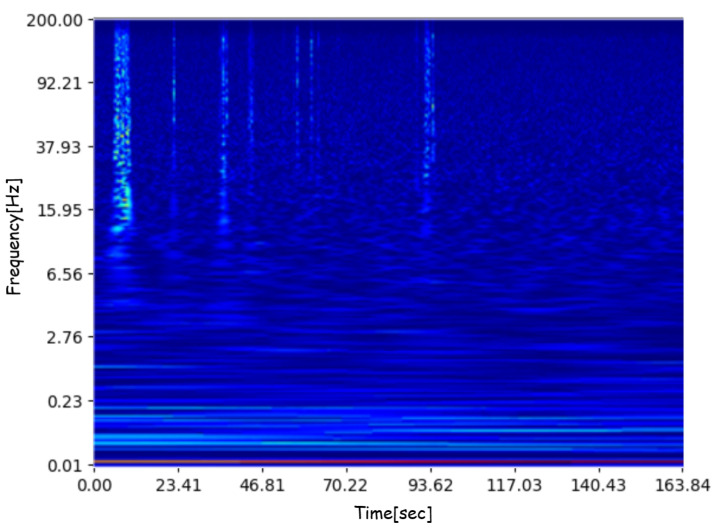
Image showing the result of SCWT function on the input signal, followed by a JET color mapping to obtain a suitable RGB image representation.

Our choice fell on SCWT transformation as it is one of the most successful time–frequency transforms available, with promising results for several tasks such as signal recovery under severe noise, voice modeling, physiological signal classification, tumor categorization, and so on [54,55,56]. In fact, besides the wavelet transform function, which is able to give an effective time–frequency signal representation [57,58], SCWT also applies a priori to sparsely localize oscillations by taking advantage of TF representation redundancies, making the assumption that components with the same instantaneous frequency can be fused together to obtain a more precise, concentrated TF representation. The main formula behind the SCWT transformation is represented in Equation (1):

(1) $$x\left(t\right)=\sum_{k=1}^{K}A_{k}\left(t\right)e^{2i\pi \phi_{k}\left(t\right)}$$

where $A_{k}$ and $\phi_{k}$ are, respectively, time-varying amplitude and phase functions. Moreover, in order to define and use the SCWT correctly, we must make some additional choices. Specifically, we need to decide on the representation of the wavelet function. In our case, we used the Morlet wavelet [59] since it is one of the most suitable wavelets for time–frequency analysis of non-stationary time-series data. We employed the logarithmic scale for the visual representation of the result, which is a gray-scale image of size 228×432×1. Finally, we applied the JET colormap function in order to expand the single image channel to three RGB channels, which is the image format most suitable for the exploitation of pre-trained deep neural networks. We used a publicly available Python library to perform the data transformation [60]. An example of the image produced by the application of the SCWT function followed by the JET colormap can be seen in Figure 3.

### 3.2. Feature Extraction and Classification

After the data processing step, we proceeded to test different pre-trained convolutional neural networks, which could serve as backbone networks in our pipeline.

Specifically, we exploited ResNet50 [61], DenseNet121 [62], and MobileNet v1 [63] architectures, chosen among others due to their ability to extract relevant visual features while having different numbers of trainable parameters and computational complexity. In fact, the MobileNet v1 model is one of the fastest networks available in the deep learning field, with a lightweight architecture made of fewer than 11 million trainable parameters. On the other side, the ResNet50 model has a number of trainable parameters equal to 40 million and represents a reliable, robust, and successful deep model widely employed as a backbone [64,65]. Finally, the DenseNet121 model is one of the most powerful (accuracy-wise) CNN architectures, with multiple residual connections among the convolutional layers, which bring the total number of trainable parameters to 14.7 million, although being heavier than the other two as it requires the most computational power and GPU memory. All these networks have been pre-trained on the ImageNet dataset [66] to obtain robust and general visual features that have been specialized through a fine-tuning training procedure on the whole network except the last fully connected layer, which has been trained from scratch to match the actual classification task. Finally, we would like to remark that our method is network-agnostic so that any kind of convolutional or transformer network can in principle be used as a backbone model for features extraction.

### 3.3. Image-Splitting

**Figure 4:**
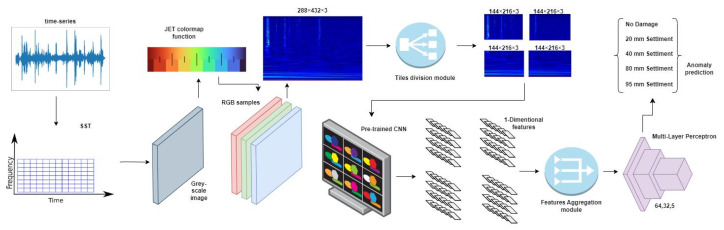
Picture showing the pipeline of the proposed method with the addition of the *image-splitting* technique.

The baseline model described in the previous paragraph is already able to produce a high accuracy; however, in order to further the method performances further, we implemented an *image-splitting* technique. It consists of the splitting of the input image into four tiles, namely the upper-right tile, the upper-left tile, the lower-right tile, and finally the lower-left tile. Figure 4 reports our proposed pipeline with the use of the image-splitting technique.

After the tiling step, each resulting image is fed to the backbone network, and the visual features of the last layer before the fully connected layer are accumulated in an aggregated vector for the final classification. In this way, the fully connected layer appointed to produce the classification receives an increased number of features, potentially improving the accuracy performances. In order to reduce the total number of experiments, we analyzed the image-splitting technique only on the best-performing network (ResNet50), but it is easily applicable on any other backbone as well without any loss of generality. However, an alternative way to increase the number of features and thus the corresponding performances are defined in the next paragraph.

### 3.4. Signal-Splitting

**Figure 5:**
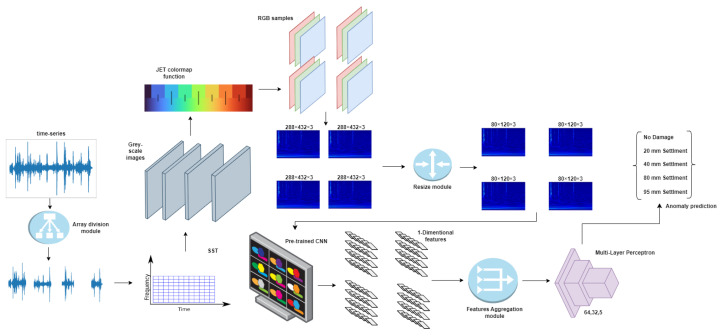
Picture showing the pipeline when using the *signal-splitting* technique.

A second option to increase the number of available features for the classification step is to split the input signal and produce the corresponding image for each sub-signal. We define this technique as *signal-splitting*. In contrast to the previous method, which divided each image into four tiles, we now divide each of the signal time-series arrays into four segments. Each segment is processed independently using the SSWT with the same parameters described in the previous sections. At this point, we again obtain four different images, each representing a part of the original time-series array. The rest of the pipeline works as before, with each image fed into the backbone network and the resulting features aggregated into a single vector for classification. The pipeline exploiting the signal-splitting technique is depicted in Figure 5.

## 4. Implementation Details

To provide a comprehensive perspective on the development of our proposed methods, we show the implementation details in this section. We introduce the dataset that we employed for validating our experiments and the task that we selected among all the possible ones on that benchmark dataset. We also report the hyperparameter settings of our implemented models and explain the metric that we adopted for characterizing and comparing the behavior of the models.

### 4.1. Dataset

In order to set up meaningful experiments we chose a dataset made of vibrational measurements acquired from a real bridge, and in particular the Z24 bridge [67]. The Z24 bridge benchmark dataset served as a test set for several solid mechanics-based and data-driven SHM damage identification techniques. It includes information of the SHM sensor from a three-span, post-tensioned concrete box girder bridge in Switzerland that has been under observation for almost a year. After nine months of routine use, during its final month of operation, the bridge was exposed to artificially produced damage. Environmental parameters were also captured concurrently with the SHM accelerometers. In [67], you can find comprehensive information about the Z24 bridge SHM program and the several performed tests. In particular, several SHM subprograms with distinct objectives were carried out on the Z24 bridge. The two primary SHM subprograms were as follows:

- A long-term continuous monitoring test for the year prior to the harm. The purpose of this test was to look at the effects of the environment on the dynamics of the bridge structure.
- A month before the bridge was destroyed, short-term progressive damage testing under various damage scenarios.

**Figure 6:**
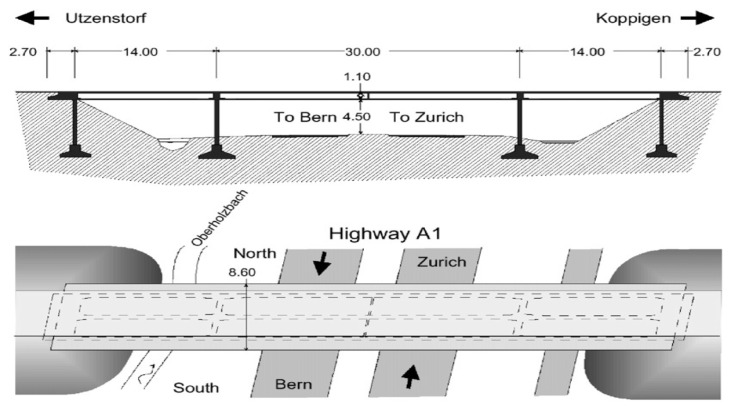
Top and cross-sectional views of Z24 bridge.

The objective of these subprograms was to investigate the dynamics of the bridge under fifteen different damage scenarios and two reference undamaged conditions. Among all the different benchmarks that can be derived from these settings, we selected one that enabled the use of produced data for a multiclass damage classification task. In particular, the bridge had nine sensor network setups for the last subprogram, each of which recorded the vibrations caused by the tests for progressive damage. Each setup employed 33 accelerometers. A representation of the top view and the cross-sectional view of the Z24 bridge can be seen in Figure 6.

We would like to remark that this dataset offers the unique possibility of testing damage detection algorithms on a real-world bridge that has been effectively damaged with several degrees of damage. However, it cannot be ruled out that some bias and coupling factors between the installed sensors and the bridge structures could make the damage detection algorithm overfit on that particular setup, instead of generalizing on the features of the damaged structures. In order to further toughen up the generalization capabilities of such methods, it is advisable to also take into consideration medium-sized, laboratory bridge models as well data coming from bridges simulated on CAD softwares [68].

### 4.2. Ambient Vibration Test vs. Forced Vibration Test

In the dataset, two types of data measurements were collected, grouped in the so-called *forced vibration test* and *ambient vibration test*. In the former, the bridge was shaken by EMPA’s two vertical shakers. The dual-channel shaker input signal was produced using the inverse FFT algorithm so that it was possible to create a force–frequency spectrum with a fairly flat frequency range from 3 to 30 Hz. The other data measurements provided are related to the ambient vibration test, which used a new data-gathering program and a measurement system that was recently installed. These measurements relied on the ambient perturbations (e.g., wind) to produce the sensor’s signals. We used the ambient vibration test measurements to perform our experiments since they can be obtained without needing expensive structures or machines to produce the signals.

### 4.3. Task and Dataset Definition: The Lowering of Pier Task

From all the damaged scenarios provided in the Z24 benchmark, we chose to test our implemented models on what we considered the most challenging multiclass classification task, based not only on the number of categories but also on the similarity between them, which differ only by the intensity of the applied damage. In particular, we chose the “lowering of pier” task, which is a multiclass classification problem with 5 different categories, as reported in Table 1.

The dataset was composed of a total of 1422 time series. Starting from this set of data, we obtained the train and test set using a 1/3 split factor, leading to a train set composed of 948 time series and a test set composed of 474 time series. We would like to remark that we built this custom train–test split because no official train–test splits were provided; as this choice is performed also by other works that propose different bridge damage identification techniques, it should be emphasized that a direct comparison between the accuracies obtained by different methods could be misleading as they are not tested on the same set of train–test data.

### 4.4. Hyperparameters

Before the conversion to temporal–frequency images, data values were normalized between 0 and 1, making use of the min–max normalization [69]. The images produced by the chosen SCWT algorithm had a shape of 288×432×3. The batch size used during the training was equal to 64. The classifier module is a simple sequence of three fully connected layers: the first one is composed of 64 units while the second has 32 units, both exploiting a LeakyReLU activation function [70]. The last fully connected layer has a number of neurons equal to the number of categories in the selected task (5) and a softmax activation function. Since the model was dealing with a multiclass classification problem, a sparse-categorical cross-entropy function was used as a loss function. As a loss optimizer, we selected the Adam optimizer [71] with an initial learning rate equal to 0.001 for a total of 40 epochs. However, the learning rate was reduced by a factor of 0.2 for the last 20 epochs. A dropout layer with a drop rate equal to 0.3 was also included in the first and second fully connected layers to reduce overfitting. For the image-splitting method, image crops were selected with an equal shape of 144×216×3. In the case of the signal-splitting method, the original time series were split into four equal segments. After the conversion to images, each image was then resized with a shape of 80×120×3, keeping the same height–width ratio as the original image. The use of a reduced resolution in both image- and signal-splitting methods enables the reduction of total GPU memory and computation; the use of the most recent GPUs could enable the implementation of our pipeline with full-resolution images.

### 4.5. Performance Metrics

In order to access the performances of the considered models, we chose as a performance metric the accuracy on the given classification task, defined as $\frac{N}{T}$, with *N* being the number of correct predictions and *T* being the total number of predictions. The reached accuracy is expressed in Section 5 as a percentage of the correct classified samples with respect to the whole test set. Moreover, we considered three additional metrics: precision, recall and F1-score.

The precision metric is defined as $\frac{tp}{tp\;+\;fp}$, where tp is the number of *true positive samples* (the samples belonging to the positive class, correctly classified as positive class) and fp is the number of *false positive samples* (the samples belonging to the negative class, incorrectly classified as positive class). The precision metric is intuitively representing the ability of the classifier to not label a negative sample as positive.

The recall metric is defined as $\frac{tp}{tp\;+\;fn}$, where fn is the number of *false negative samples* (the samples belonging to the positive class, incorrectly classified as negative class). This metric is intuitively representing the ability of the classifier to find all the positive samples.

Finally, F1-score is defined as $\frac{tp}{tp\;+\;0.5\;(fp\;+\;fn)}$. the F1-score is actually calculating the harmonic mean of the precision and recall scores. The F1-score measures how much precision and recall scores deviate from each other, as more differences between precision and recall values correspond to worse F1-score values.

In the case of our multi-category classification task, these additional metrics were calculated with a one-versus-all scheme, with the mean values across all the categories being reported in the results tables.

Finally, we also reported the number of trainable parameters for each method, which is a fair complexity indicator of the considered models.

## 5. Results

In this section, we evaluate the performance of our proposed models together with several machine learning and deep learning methods that are suitable for this classification task, both in the original 1D domain and in the transformed 2D domain. Moreover, we tested the performances of several different networks when used as the backbone in our method. In fact, Table 2 shows a comparison between ResNet50, MobileNet v1, and DenseNet121 networks when used as a backbone. The ResNet50 model achieved the highest accuracy of 97.08%; almost 2% more accuracy than the MobileNet v1 architecture and almost 7% more than the DenseNet121 one. We hypothesize that this behavior is accounted by the highest number of trainable parameters of the ResNet50 network; however, the slight decrease in the performance of MobileNet v1 still makes it suitable for resource-constrained settings, such as performing the classification procedure on site exploiting embedded, low-powered devices (https://coral.ai/products/dev-board/, accessed on 3 May 2023). Such behavior is confirmed by the precision, recall and F1-score metrics, which exhibit the highest values in the case of ResNet50, with the three values equal to each other, which corresponds to the number of false positives equal to the number of false negatives for this network. However, as can be noticed also from the values of precision, recall and F1-score for MobileNet v1 and DenseNet121, our method does not have any preferred category for any considered backbone.

In Table 3, instead, we show the impact on the performances of the image-splitting and signal-splitting techniques. For brevity, we applied those techniques to our model using the backbone with the best accuracy (ResNet50). The accuracy increase of image-splitting and signal-splitting techniques were, respectively, 0.4% and almost 0.5%; although the absolute accuracy increase seems to be limited, they are both able to bring the error from around 3% (one misclassification out of 33) to 2.5% (one misclassification out of 40), which is a considerable improvement on a critical task such as damage detection on bridges. The other considered metrics showed the highest values in the case of signal-splitting, confirming the validity of this technique, with all metrics showing similar values as the proposed method did not overfit on a specific category over the others. Finally, we can conjecture that the increase in performance (similar in the two methods) is probably due to the same increase in the total number of visual features fed to the classification layer.

In Table 4 we show a comparison between our proposed models (with and without the image- or signal-splitting techniques) and some popular baseline models applied to the same task. In particular, our models have been compared with a simple multi-layer perceptron network, which works on original time-series data, and on three deep models, called 1DCNN, LSTM, and TDNN. The 1DCNN [48] model has been developed specifically for damage classification on the Z24 bridge. It works by applying a 1D convolutional neural network on 1D signals (time series) coming from sensors and it implements a custom signal window technique; these reasons make it the ideal candidate for comparison with our method. Unfortunately, the train and test split performed by the authors are not provided, making the comparison data biased and possibly unfair. Because of that limitation, we report both the performances as shown in the original paper (last four rows of Table 4) and the performances obtained by running that method on our train and test splits (first four rows of Table 4). The second compared deep model [72], referred to as LSTM, takes advantage of the time relations between consecutive samples in a time series, producing accurate, temporally related features, while implementing a custom window function as well, in order to feed data to a long short-term memory module. As in the previous case, we report both the original results as well as the results of a run on our defined train and test data split. Finally, the TDNN model [73] works by extracting context from the time series using a time-delay neural network, pre-trained on audio signals. We report only the accuracy from the original paper as the model source code is not publicly available.

Looking at the reported performances, our method with the signal-splitting boost produces the highest accuracy, with an improvement of 3.5% with respect to the original LSTM model results and more than 6.5% with respect to the same model running on our set of data. All the other compared deep methods are lagging behind, with an accuracy ranging from 83% to 86%, making them unsuitable for a critical task such as damage identification, which usually requires a very high classification accuracy. The shallow method based on multi-layer perceptron clearly fails to provide a good classification system, with an accuracy value of 72%.

Besides the splitting methods that act as a booster module, our model’s superior performance lies in the use of a proficient time–frequency transformation and a CNN pre-trained on ImageNet, enabling the exploitation of robust and general visual features that are suitable for classifying various damage scenarios in the data.

Looking at all the values of precision metrics, which show small deviation with respect to the corresponding recall, we can deduct that for all the considered metrics the numbers of false positives and false negatives are quite similar, and that the classes balance in the Z24 bridge dataset is helping the classifiers to not overfit on a single category.

## 6. Conclusions

In this work, we propose a model for damage identification on bridges. Our proposed method achieves an accuracy of 97.5%, making it the most accurate method when compared with state-of-the-art techniques on the same dataset. Our results show that the ResNet50 model is highly effective when exploited as a backbone, but that other networks such as MobileNet v1 are still exploitable for high performances in low-resource scenarios. Additionally, we found that the image-splitting and signal-splitting methods can improve the accuracy of the overall classification. The other metrics, with their high and similar values, confirm the capability of our method to perform effective classification in all the five categories without sensible distinction between false positives and false negatives. Above all, we demonstrate the power of temporal–frequency transformation methods such as SCWT for such tasks, as they enable the use of pre-trained deep neural networks for the development of robust visual features able to distinguish different damage scenarios. In future works, we plan to test other architectures based on different paradigms, such as the transformer networks, comparing them with the traditional CNN to understand the impact of the transformer module on the anomaly detection task. Moreover, we plan to evaluate our algorithm on other bridge datasets with different types of sensors and anomalies. Another possible line of research is to explore scalable and distributed solutions that can handle large-scale and high-frequency sensor data using edge computing and cloud computing technologies. Finally, we believe that the presented model can be effectively employed also in other damage classification tasks, for example on different structures with respect to the bridges, which will be a subject of future research.

## Figures and Tables

**Table 1:** Considered damage scenarios for the classification task and the relative number of samples (time series) associated with them.

Damage Scenario	Number of Time Series	Class Label
Undamaged	291	0
Lowering of pier, 20 mm	258	1
Lowering of pier, 40 mm	291	2
Lowering of pier, 80 mm	291	3
Lowering of pier, 95 mm	291	4

**Table 2:** Table showing the performances reached by the proposed method when exploiting different backbone architectures. The first four rows report the test accuracy together with precision, recall and F1-score, while the last row shows the number of trainable parameters of the considered backbone network. The best results are reported in bold.

	ResNet50	MobileNet v1	DenseNet121
**Accuracy**	**97.08%**	95.36%	90.21%
**Precision**	**97.22%**	94.41%	87.14%
**Recall**	**97.22%**	94.06%	87.08%
**F1-score**	**97.22%**	94.12%	86.92%
**Parameters**	40,105,093	**10,898,885**	14,707,525

**Table 3:** Table showing the test accuracy, precision, recall, and F1-score reached by the baseline model and the two splitting techniques, when using the same backbone (ResNet50). The best results are reported in bold.

	ResNet50	Image-Splitting	Signal-Splitting
**Accuracy**	97.08%	97.47%	**97.50%**
**Precision**	97.22%	97.39%	**97.77%**
**Recall**	97.22%	97.17%	**97.34%**
**F1-Score**	97.22%	97.27%	**97.51%**

**Table 4:** Table showing the performances produced by our methods compared with other techniques. The metrics without the * character refer to the results of the methods as published in their respective works, while the metrics with the * character refer to the same methods running on our train and test data splits. The best results are reported in bold.

	ResNet50	Image-Splitting	Signal-Splitting	MLP	1DCNN	LSTM	TDNN
Accuracy *	97.08%	97.47%	**97.50%**	72.05%	86.28%	90.92%	
Precision *	97.22%	97.39%	**97.77%**	73.17%	88.18%	89.21%	
Recall *	97.22%	97.17%	**97.34%**	71.31%	86.55%	88.74%	
F1-score *	97.22%	97.27%	**97.51%**	71.42%	86.79%	88.61%	
**Accuracy**					85%	94%	83.08%
**Precision**					86%	95%	
**Recall**					85%	94%	
**F1-score**					85%	94%	

## Data Availability

Our work is validated on the Z24 bridge benchmark dataset, which is available upon request at the following address: https://bwk.kuleuven.be/bwm/z24, accessed on 1 February 2023.

## Abbreviations

The following abbreviations are used in this manuscript:

SHM	Structural health monitoring
SST	Synchrosqueezing transform
SDD	System damage detection
ML	Machine learning
CNN	Convolutional neural networks
ANN	Artificial neural networks
TF	Time–frequency
STFT	Short-time Fourier transform
WT	Wavelet transform
CWT	Continuous wavelet transform
VDD	Vibration-based damage detection
AR	Auto-regressive
ARMA	Auto-regressive moving-average
ARX	Auto-regressive exogenous input
CCWT	Continuous Cauchy wavelet transform
DL	Deep learning
FCN	Fully convolutional networks
RNN	Recurrent neural networks
Re LU	Rectified linear unit
GPU	Graphics processing unit
LSTM	Long short-term memory
RGB	Red, green and blue
EMPA	Swiss Federal Laboratories for Materials Testing
FFT	Fast Fourier transform
TDNN	Time-delay neural network
MLP	Multi-layer perceptron
